# The Electrochemical Oxidation of Hydroquinone and Catechol through a Novel Poly-geminal Dicationic Ionic Liquid (PGDIL)–TiO_2_ Composite Film Electrode

**DOI:** 10.3390/polym11111907

**Published:** 2019-11-19

**Authors:** Yanni Guo, Deliang He, Aomei Xie, Wei Qu, Yining Tang, Lei Zhou, Rilong Zhu

**Affiliations:** 1 State key Laboratory of Chemo/Biosensing and Chemometrics, College of Chemistry and Chemical Engineering, Hunan University, Applied Chemistry, Changsha 410082, China; gyn0303@hnu.edu.cn (Y.G.); qw779470790@163.com (W.Q.); yiningt@163.com (Y.T.); zl3139552204@hnu.edu.cn (L.Z.); 2 College of Marxism, Jishou University, Jishou 416000, China; xieaomei@163.com

**Keywords:** nano-TiO_2_, poly-geminal dicationic ionic liquid, hydroquinone, catechol

## Abstract

A novel poly-geminal dicationic ionic liquid (PGDIL)-TiO_2_/Au composite film electrode was successfully prepared by electrochemical polymerization of 1,4-bis(3-(m-aminobenzyl)imidazol-1-yl)butane bis(hexafluorinephosphate) containing polymerizable anilino groups in the electrolyte containing nano-TiO_2_. The basic properties of PGDIL–TiO_2_/Au composite films were studied by SEM, cyclic voltammetry, electrochemical impedance spectroscopy, and differential pulse voltammetry. The SEM results revealed that the PGDIL–TiO_2_ powder has a more uniform and smaller particle size than the PGDIL. The cyclic voltammetry results showed that the catalytic effect on electrochemical oxidation of hydroquinone and catechol of the PGDIL–TiO_2_ electrode is the best, yet the *R*_ct_ of PGDIL–TiO_2_ electrode is higher than that of PGDIL and TiO_2_ electrode, which is caused by the synergistic effect between TiO_2_ and PGDIL. The PGDIL–TiO_2_/Au composite electrode presents a good enhancement effect on the reversible electrochemical oxidation of hydroquinone and catechol, and differential pulse voltammetry tests of the hydroquinone and catechol in a certain concentration range revealed that the PGDIL–TiO_2_/Au electrode enables a high sensitivity to the differentiation and detection of hydroquinone and catechol. Furthermore, the electrochemical catalytic mechanism of the PGDIL–TiO_2_/Au electrode was studied. It was found that the recombination of TiO_2_ improved the reversibility and activity of the PGDIL–TiO_2_/Au electrode for the electrocatalytic reaction of HQ and CC. The PGDIL–TiO_2_/Au electrode is also expected to be used for catalytic oxidation and detection of other organic pollutants containing –OH groups.

## 1. Introduction

Poly ionic liquids (PILs) are a kind of functional polymer material that contain at least one ion center in a polymer chain and a repeating unit similar to a common ionic liquid (IL) structure; they combine the properties of polymers and ionic liquids, and show are in the foreground of applications for ionic conductors, adsorption and separation, dispersants, and catalysts [1,2,3,4]. Therefore, the research on the preparation and performance of PILs has aroused wide interest and concern in recent years [5,6].

The dicationic ionic liquids (DILs) are considered to be a combination of three structural moieties: (1) cationic head groups; (2) a linkage chain (also called a spacer); and (3) counter anions. DILs can be classified as either homoanionic or heteroanionic, which can further be categorized as symmetrical or asymmetrical. Homoanionic dicationic ionic liquids are typical DILs that consist of a dication and two identical anions. Symmetrical or geminal dicationic ILs (GDILs) can be synthesized by joining two of the same cations, such as imidazolium or pyrrolidinium, and may contain a cyclic or aliphatic chain via either a rigid or a flexible spacer. A common spacer is an alkyl chain [7,8]. Armstrong et al. [9] studied the relationship between the structure and properties of DILs by synthesizing 39 imidazolium-based and pyrrolidinium-based DILs. The head groups were linked with an alkyl chain (from three to 12 carbons long), and hence reacted with four different traditional anions. The thermal stability of these ILs was found to be in the range of −4 to > 400 °C, which is greater than that of most traditional monocationic ILs. GDILs have more unique physical and chemical properties and solvation characteristics, and can be used as separation materials [10], surfactants [11], and catalyst candidates [12,13], although they have not yet been studied heavily [14,15]. The same is true of poly-GDILs (PGDILs).

As is known, nano-TiO_2_ has better chemical properties and photon characteristics due to its good absorbability and lower electron/hole recombining rate, and can be used as a new kind of electrical catalyst material [16,17,18]. There have been many reports on the application of nano-TiO_2_ composite polymers in the electrocatalysis of organic materials [19], including a study by the present research group [20]. Aniline is a kind of monomer that is easy to polymerize. Polyaniline is an important conductive polymer [21,22,23] that can be used as anode or cathode material with electrocatalytic function due to its excellent electrical and electrochemical properties [24]. The combination of polyaniline and nano-TiO_2_ can not only effectively inhibit the agglomeration of TiO_2_ nanoparticles, but also improve the physical and chemical properties of composites, and may be widely applied in the electrochemical catalysis field [20,25,26].

Hydroquinone (HQ) and catechol (CC) are important phenolic compounds that are widely used as basic raw materials in the organic chemical, agriculture, and medicine industries, among others [27]. They are typical and important electro-active molecules in fundamental electrochemical research. Therefore, it is of great significance to establish a high-sensitivity detection method for HQ and CC in the fields of environmental monitoring and food inspection. Moreover, the chemical structures and the physicochemical properties of HQ and CC are very similar, and they are, therefore, difficult to distinguish [28]. Current methods for the determination of HQ and CC include fluorescence [29], the electrochemical method [30], the photometric method [31], and high-performance liquid chromatography [32]. The electrochemical method is highly valued because of its versatility, simple operation, easy automation, and environmental compatibility. In electro-catalytic reactions, in which an electrode is an electrical catalyst, different electrode materials can change the electrochemical reaction rate by different magnitudes, so new and efficient catalytic electrode materials have always been a focus of related research.

The present research group previously successfully synthesized 1,4-bis(3-(m-aminobenzyl)imidazol-1-yl)butane bis(hexafluorinephosphate) ([C_4_(m-ABIM)_2_][PF_6_]_2_), which is a novel GDIL containing anilino groups, and poly-[C_4_(m-ABIM)_2_][PF_6_]_2_ (PGDIL) with a polyaniline-like structure was prepared by electro-polymerization [33]. In this study, a novel PGDIL–TiO_2_ composite was prepared via electrodeposition on an Au electrode surface, and the electrochemical redox behaviors of HQ and CC were respectively investigated via the cyclic voltammetry (CV) and differential pulse voltammetry (DPV) methods. It was expected that the PGDIL–TiO_2_ composite film would have the advantages of both PGDIL and TiO_2_.

## 2. Experimental

### 2.1. Main Reagents and Instrumentation

The primary reagents used in the experiments, [C_4_(m-ABIM)_2_][PF_6_]_2_ ([33]), sodium dihydrogen phosphate, disodium hydrogen phosphate, potassium chloride, hydroquinone, and anatase nano-TiO_2_ powder (10–25 nm), were of analytical grade, and were used as received. The water used in the experiment was double distilled water.

The instruments used in the experiments were an automatic double distilled water distillation apparatus D1810C (Shanghai Asia-Pacific Glass instrument Company, Shanghai, China); a heat-collecting, constant temperature magnetic heating stirrer CJJ 78-1 (Zhengzhou Greatwall Scientific Industrial and Trade Co., Ltd., Zhengzhou, China); a rotary evaporator R.1002 (Zhengzhou Greatwall Scientific Industrial and Trade Co., Ltd., Zhengzhou, China); an ultrasonic cleaner SK5200HP (Shanghai Kudos Ultrasonic instrument Co., Ltd., Shanghai, China); an infrared spectrophotometer TJ270-30 (A) (Tianjin Jinwei Electronic Instrument Co., Ltd., Tianjin, China ); and an electrochemical workstation IviumStat (Ivium Technologies BV, Eindhoven, Netherlands).

### 2.2. Synthesis of Poly-[C_4_(m-ABIM)_2_][PF_6_]_2_–TiO_2_ Composite Film

An electrochemical tri-electrode system was introduced for electropolymerization. An Au electrode, platinum wire, and Ag/AgCl electrode were used as the working electrode, counter electrode, and reference electrode, respectively. Prior to the surface modification, Au electrodes were separately polished by alumina particles with diameters of 1, 0.3, and 0.05 μm to obtain mirror-like surfaces. After successive sonication in ethanol and double distilled water successively for 10 minutes (5 minutes each), the polished electrodes were rinsed with water and then dried with an air blower. All electrochemical experiments were carried out at room temperature under a nitrogen atmosphere.

A 0.5 mg/mL TiO_2_ uniform suspension solution (prepared via the ultrasonic dispersion of 10 mg nano-TiO_2_ power in 20 mL anhydrous ethanol containing 5 wt % nafion) was mixed with an equal volume of 0.05 mol/L acetonitrile solution of GDIL (containing 0.1 mol/L sodium perchlorate) via ultrasonic agitation. Then, the Au electrode as the working electrode was placed in this mixture to prepare the PGDIL–TiO_2_ composite film by means of chronoamperometric polymerization at a constant potential of 1.1 V for 300 s. As control subjects, PGDIL film without TiO_2_ was also prepared in the same chronoamperometric polymerization conditions_,_ and TiO_2_ film was prepared by dropping 10 μL 0.5 mg/mL TiO_2_ uniform suspension solution onto the bare gold electrode.

**Figure 1:**
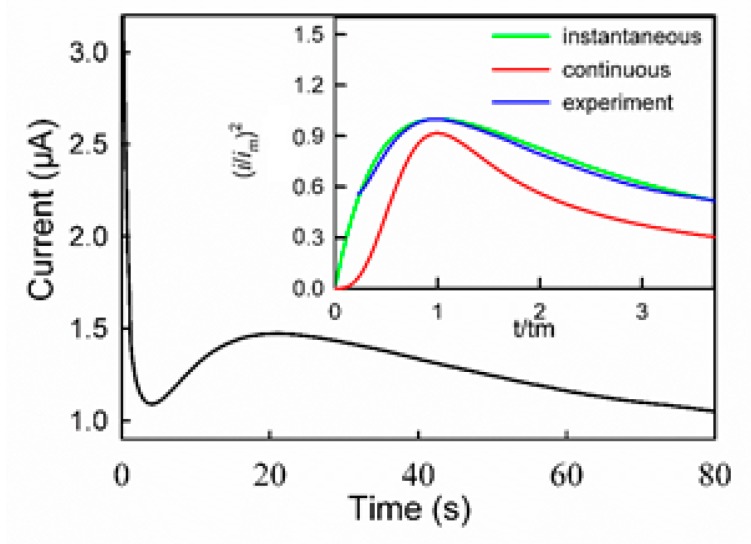
Current versus time transient during the potentiostatic electropolymerization of geminal dicationic ionic liquid (GDIL)–TiO_2_ at a potential of 1.1 V. Inset: comparison of the experiment curve with theoretical curves from the Scharifker–Hills model.

Chronoamperometry is an important diagnostic technique for the initial stage of electro-crystallization [34]. Figure 1 sows a current transient (CTT) recorded during the polymerization process of GDIL-TiO_2_ at 1.1 V. The electropolymerization process should be hardly affected by other reactions. The CTT can be divided into three regions, which is consistent with the aggregation polymerization process of GDIL [33]. In the first region (*t* < 2 s), the decrease in oxidation current is related to charging of the double layer due to the specific adsorption of GDIL on the Au electrode. The second region (2 s < *t* < 21 s) corresponds to the increase in the oxidation current up to a maximum, which is typical of nucleation and growth processes. The third region (*t* > 21 s) corresponds to the decrease in the oxidation current, which is typical of a diffusion-controlled process. An analysis of the CTT was performed by fitting the experimental data to a dimensionless theoretical curve for crystal nucleation and diffusion-controlled growth in three dimensions (3D), as proposed by Scharifker and Hills [35]. The instantaneous and progressive theoretical transients are given by Equations (1) and (2), respectively:

(1) $$\frac{i^{2}}{i_{m}^{2}}=\frac{1.9542}{t/t_{m}}{\{1-\exp[-1.2564(t/t_{m})]\}}^{2}$$

(2) $$\frac{i^{2}}{i_{m}^{2}}=\frac{1.2254}{t/t_{m}}{\left\{1-\exp[-2.3367{\left(t/t_{m}\right)}^{2}]\right\}}^{2}$$

where *i_m_* and *t_m_* represent the maximum current density and its corresponding time, respectively.

The experimental data were fitted as shown in the inset of Figure 1. The instantaneous nucleation is dominant at the oxidant peak potential in the electropolymerization of GDIL-TiO_2_, because the experimental curve conforms to the theoretical curve of the instantaneous nucleation model.

### 2.3. Electrochemical Performance Tests

All electrochemical treatments were conducted at room temperature. in 0.1 mol/L KCl containing K_3_[Fe(CN)_6_]/K_4_[Fe(CN)_6_] (5.0 × 10^−3^ mol/L each). Electrochemical impedance spectra (EIS) were tested in the frequency range between 100,000 and 0.01 Hz at an alternating current voltage amplitude of 5 mV. Cyclic voltammetry (CV) measurements were performed at a scan rate of 0.05 V/s in the scanning range of −0.2–0.6 V.

The electrochemical behaviors of HQ and CC on the PGDIL–TiO_2_/Au, PGDIL/Au, TiO_2_/Au, and Au electrodes were preliminarily investigated by CV and differential pulse voltammetry (DPV) methods, respectively. Platinum wire and Ag/AgCl electrode were used as the counter electrode and reference electrode, respectively. The buffer solutions used in all electrochemical experiments were pH = 7 NaH_2_PO_4_–Na_2_HPO_4_ (PBS). For continuous determination, the HQ and CC adsorbed on the electrode surface could be removed by CV for 10 cycles at a 0.05 V/s scan rate in the buffer solution.

## 3. Results and Discussion

### 3.1. Polymerization Mechanism of PGDIL–TiO_2_ Electrode

**Figure 2:**
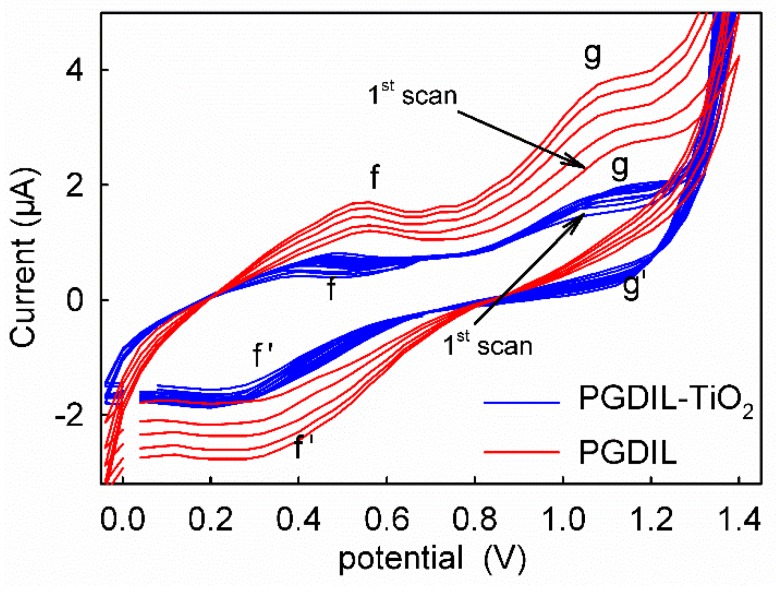
The cyclic voltammetry curve of GDIL. The arrows were the 1st scan of the CV curves of GDIL and GDIL–TiO_2_ on the Au electrode. g and g’ were the oxidation and reduction peak of the monomer, respectively. f and f’ were the oxidation and reduction peak of the polymer molecular chain, respectively.

The CV curves of GDIL–TiO_2_ on the Au electrode are presented in Figure 2. In the first scan process, the apparent oxidation peak g could be found at the anode 1.0 V, and was induced by oxidation of the anilino-groups of GDIL to a cation radical. The reduction peak g*’* of the cation radical appeared in the process of the back sweep cathode, and decreased with the scanning. This indicates that with the continuation of the reaction, the cation radical generated was continuously consumed, and the polymerization generated PGDIL. The current of the redox peak f–f*’*, which is the redox peak pair for the formation of a chain-type polymer reaction, increased with the polymerization, and finally stabilized. The CV curves of GDIL on the Au electrodes also have the appearance of this redox peak pair, which proves that f–f*’* peak is the redox peak pair of polymerization. However, the polymerization process of GDIL on the Au electrode did not contain this reduction peak of the cation radical. It is possible that the cationic base generated was more active and reacted immediately; this is also illustrated by the peak current response of GDOL on the Au electrode, which is significantly greater than the peak current response of GDIL–TiO_2_. It can be seen that the electropolymerization mechanism of GDIL–TiO_2_ and GDIL were the same, but the polymerization rate of GDIL–TiO_2_ was slower than that of GDIL [33].

### 3.2. SEM Characterization of PGDIL–TiO_2_ and PGDIL

The PGDIL–TiO_2_ film formed on the surface of the Au electrode was scraped down and dissolved in anhydrous ethanol. The ultrasonic shock made it disperse evenly and then remain stationary. The clear droplets were absorbed into the copper sheet after treatment.

**Figure 3:**
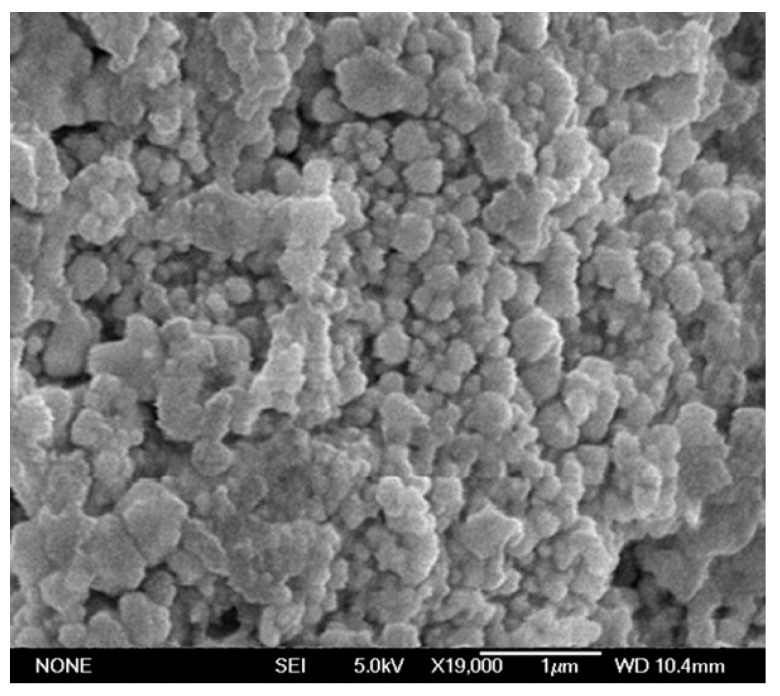
SEM image of PGDIL–TiO_2_ powder.

Figure 3 shows the scanning electron micrograph of the PGDIL–TiO_2_ powder. As shown in the images, the PGDIL–TiO_2_ powder was composed of homogeneous, stacked, spherical particles, which is similar in shape to the PGDIL powder. Additionally, the PGDIL–TiO_2_ powder had a more uniform and smaller particle size than the PGDIL [33]. This is because, at the beginning of electropolymerization, TiO_2_ nanoparticles adsorbed on the surface of Au electrode had a good promoting effect on the electropolymerization of GDIL with anilino groups because its O-vacancy can adsorb GDIL molecules, which forms the core of polymerization. The growth process of PGDIL–TiO_2_ particles was slow and uniform, and the resulting particles were smaller, and the film layer was thicker, than that of PGDIL. This is consistent with the CV results.

### 3.3. EIS Characterization of PGIL–TiO_2_/Au, TiO_2_/Au, and Bare Au Electrode

**Figure 4:**
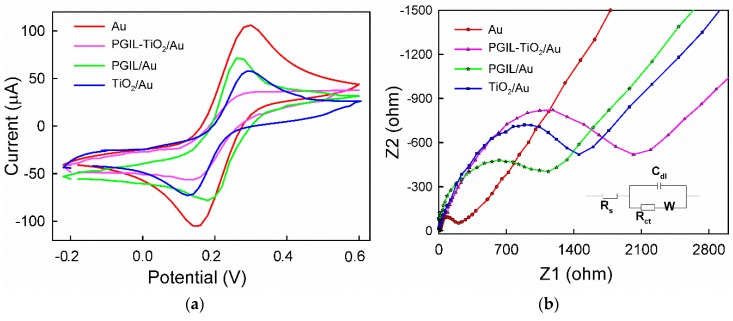
(**a**)The cyclic voltammetry curves and (**b**) the EIS plots of PGDIL–TiO_2_/Au, PGDIL/Au, TiO_2_/Au, and Au in the presence of 5.0 mmol L^−1^ Fe(CN)_6_^3−/4−^ with 0.1 mol L^−1^ KCl as the electrolyte. The illustration equivalent circuit.

The conductivity of the electrode can be judged by the redox reaction of potassium ferricyanide solution on the electrode. The stronger the conductivity of the electrode, the stronger the redox reaction of potassium ferricyanide, and the greater the redox current value, and vice versa. Figure 4a presents the cyclic voltammetry curves of the PGDIL–TiO_2_/Au, PGDIL/Au, TiO_2_/Au, and Au electrodes in 5 mmol/L Fe(CN)_6_^3−/4−^ solution containing 0.1 mmol/L KCl. From largest to smallest, the redox peak current values were Au > PGDIL > TiO_2_ > PGDIL–TiO_2_ [36].

Electrochemical impedance spectroscopy (EIS) can be used to characterize the surface electron transfer of different modified electrodes. In the resulting graph of the data (Figure 4b), the linear part indicates a diffusion-controlled process at low frequency, and the semicircle part is consistent with the electron transfer resistance at a high frequency; together, they make up the EIS results in a typical Nyquist plot. The diameter of the arc represents the charge-transfer resistance (*R*_ct_) on the surface of the electrode. The impedance is mainly composed of film resistance, ion transmission resistance in the film, and double layer capacitance at the interface between the film and the solution, which formed a large capacitive arc. It can be seen from Figure 4b that the conductivity of each electrode from smallest to largest was PGDIL–TiO_2_ < TiO_2_ < PGDIL < Au. Although the conductivity of PGDIL was inferior to that of the metal Au, it was superior to that of TiO_2_, which may be related to the low electrical conductivity of the polyaniline-like material itself. In addition, it is also possible that the PGIL film with a positive charge adsorbed part of the Fe(CN)_6_^3−/4−^, resulting in the generation of an electrostatic repulsion between the surface of the electrode material and the ions in the bulk solution; thus, there was not a significant difference in concentration between the electrode surface and the bulk solution, which prevented the reaction of Fe(CN)_6_^3−/4^ on the electrode surface [37]. The conductivity of PGDIL–TiO_2_ was worse than that of TiO_2_ and PGDIL because it included not only the resistance of TiO_2_, but the resistance of PGDIL. In the low frequency region, the PGDIL–TiO_2_ composites presented a straight line close to 45°, and the length of the Warburg curve was smaller than that of the PGDIL, which demonstrates that the electrolyte ions had a better rapid diffusion and transfer ability, and lower interfacial resistance.

### 3.4. CVs of HQ and CC on Different Modified Electrodes

**Figure 5:**
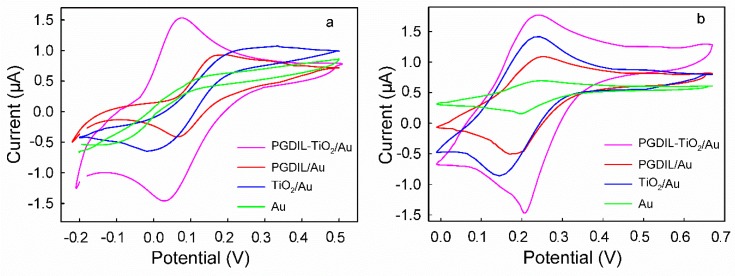
The CV results of (**a**) 0.1 mmol/L HQ and (**b**) 0.05 mmol/L CC at different modified electrodes. The scan rate was 0.1 V/s.

The CV results of 0.1 mmol/L HQ and 0.05 mmol/L CC on different modified electrodes are shown in Figure 5. The related electrochemical parameters are listed in Table 1. HQ and CC are composed of one benzene ring and two hydroxyl groups, but the CVs are slightly different due to the different hydroxyl positions. The charge density of *p*-hydroxyl group in benzene ring is higher than that in *ortho*-position, and is the lowest in *meso*-position. Because of the higher charge density, the anodic peak potential (*E*_pa_) of HQ is lower than that of CC. On the contrary, the lower the charge density, the easier it can be reduced, so the reduction peak potential (*E_pc_*) of CC is higher than that of HQ [38].

It is demonstrated by the CV results that there was no significant redox peak current response to either HQ (Figure 5a) or CC (Figure 5b) on the Au electrode, whereas a pair of obvious redox peaks appeared on the CVs of the PGDIL–TiO_2_/Au electrode of both HQ (Figure 5a) and CC (Figure 5b) solutions. The peak potential difference (Δ*E*_p_) between the anodic and cathodic peaks on the PGDIL–TiO_2_/Au electrode was smaller than that for the PGDIL/Au and TiO_2_/Au electrodes (Table 1), indicating that the reversibility of the electrochemical reaction was improved. We propose that the electrochemical processes of HQ and CC on the PGDIL–TiO_2_/Au electrode were quasi-reversible because the cathodic current (*I*_pc_) and anodic peak current (*I*_pa_) were near equal. On the other hand, the peak currents were much higher compared with those on the PGDIL/Au and TiO_2_/Au electrodes. HQ exhibited a peak current of 1.533 μA on the PGDIL–TiO_2_/Au electrode, which is 1.66 times higher than that on the PGDIL/Au electrode (0.926 μA). In contrast, the peak current of CC on the PGDIL–TiO_2_/Au electrode was 1.771 μA, exhibiting an increase of 1.62 times that on the PGDIL/Au electrode (1.090 μA). The increasing current signals and minimization of over potentials confirms that the PGDIL–TiO_2_/Au electrode has high electrocatalytic activity for electrochemical oxidation of CC and HQ.

According to the characteristics of a reversible electrode reaction [39], at 25 °C,

(3) $$\Delta E_{p}\left(\mathrm{mV}\right)=22.5\mathrm{RT}/nF=59/n\left(\mathrm{mV}\right)$$

The electron transfer number *n* of the electrochemical reaction can be obtained from the formula. It can be determined that the reaction processes of HQ and CC on the PGDIL–TiO_2_/Au electrode are two electron-involved quasi-reversible reactions. According to the experimental results, the possible electrocatalytic redox mechanism of HQ and CC can be inferred from the relevant literature [40].

**Scheme 1:**
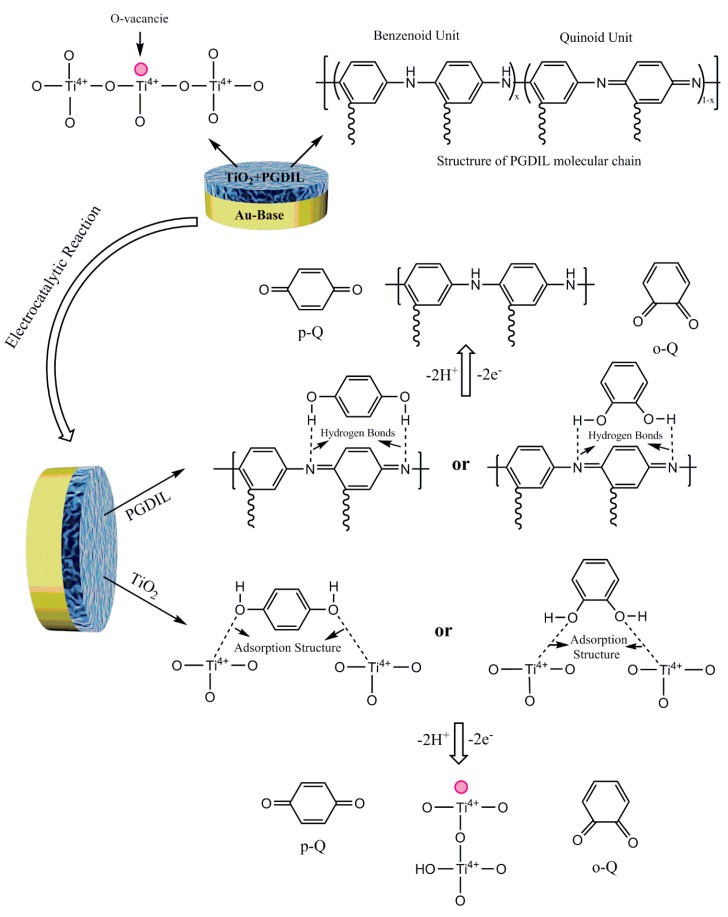
Structure of PGDIL–TiO_2_ and the oxidation processes of HQ and CC.

As shown in Scheme 1, the structure of PGDIL is a mixture of benzenoid and quinoid units [33]. In the electrocatalytic oxidation system, due to the polyaniline-like structure in the structure of the PGDIL–TiO_2_ electrode, the imino nitrogen atoms (–N=) in the conjugated quinoid moieties of polyaniline-like structure will interact with the hydroxyl groups (–OH) in HQ or CC through hydrogen bonds, accelerating the oxidation of HQ or CC [41,42]. First, CC in the solution was absorbed on the surface of the PGDIL–TiO_2_ electrode and formed two hydrogen bonds with the two hydrogen atoms of imino on the polyaniline-like structure. Then, two single electron transition processes occurred. As shown in Scheme 1, the reacting center itself is based on the hydrogen bonds, or, better yet, a hydrogen bridge that regulates the redox state of quinone (catechol or intermediate product) on the PGDIL–TiO_2_ electrode. The phenolic hydroxyl groups of HQ or CC can be easily adsorbed on the O-vacancy of the TiO_2_-anatase surface to form an adsorption structure, and, the H atoms on the phenolic hydroxyl group can be easily moved to the O atom surface at the adjacent position via hydrogen transfer. Finally, the corresponding benzodiquinone was formed. This may also explain why, although its catalytic ability was better, the *R*_ct_ value of the TiO_2_/Au electrode was higher than that of PGDIL (as shown in Figure 4 and Figure 5) [43,44]. In addition, there is a certain synergistic effect between TiO_2_ and PGDIL. Due to the electrocatalytic reaction, the quinoid units of PGDIL became benzenoid units and lost their oxidative activity, while the lone pair electrons on the N atom ($–\ddot{N}H–$) in benzenoid units occupied the O-vacancy of TiO_2_ to be restored by dehydrogenation to form quinoid units again. Therefore, the PGDIL–TiO_2_/Au electrode has higher electrocatalytic activity for electrochemical oxidation of CC and HQ than the PGDIL/Au and TiO_2_/Au electrode.

**Figure 6:**
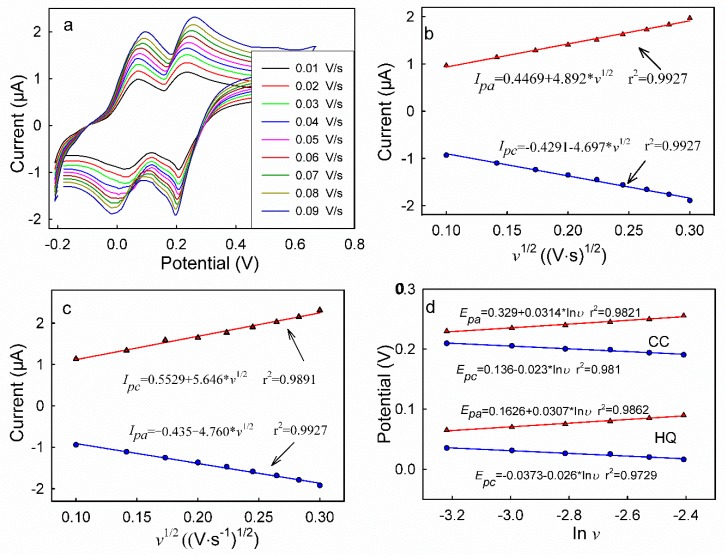
(**a**) The CV curves of HQ and CC on the PGIL–TiO_2_/Au electrode at different scan rates (0.01, 0.02, 0.03, 0.04, 0.05, 0.06, 0.07, 0.08, and 0.09 V/s). (**b**) The relationship between the redox peak current (*I*_p_) of HQ and scan rates (*v*). (**c**) The relationship between redox peak current (*I*_p_) of CC and scan rates (*v*). (**d**) The relationship between *E*_p_ and scan rates (*v*).

Cyclic voltammetry at different scan rates (0.01–0.09 V/s) was employed to further study the conduction characteristics of HQ and CC on the PGDIL–TiO_2_/Au composite film modified electrode in 0.1 mmol/L HQ and 0.05 mmol/L CC. As shown in Figure 6a, with the increase of scan rate, the anodic peak currents of HQ and CC increased, and as the anodic peak potential shifted positively, the reduction peak currents increased, and the reduction peak potential shifted negatively; these findings further prove that the redox reactions of HQ and CC are quasi-reversible processes. Both the anodic peak current value (*I*_pa_) and the cathodic peak current value (*I*_pc_) are linearly related to *υ*^1/2^ (Figure 6b,c) under the given conditions. These results indicated that the electrode reactions of CC and HQ on PGDIL–TiO_2_/Au were typical diffusion-controlled process.

As presented in Figure 6d, the anodic peak potential (*E*_pa_) and the cathodic peak potential (*E*_pc_) of HQ and CC are linearly related to ln*υ* when the ∆*E*_p_ value of HQ is greater than 32 mV, and when the *∆E*_p_ of CC is greater than 30 mV, it is in accordance with the Laviron equation [45]:

(4) $$E_{\mathrm{pc}}=E^{\theta }+\frac{\mathrm{RT}}{\alpha nF}\mathrm{In}\frac{\mathrm{RT}\kappa_{s}}{\alpha nF}-\frac{\mathrm{RT}}{\alpha nF}\ln\nu$$

(5) $$E_{\mathrm{pa}}=E^{\theta }+\frac{\mathrm{RT}}{(1-\alpha )\mathrm{nF}}\mathrm{In}\frac{\mathrm{RT}\kappa_{s}}{(1-\alpha )nF}+\frac{\mathrm{RT}}{(1-\alpha )nF}\ln v$$

where *E*_pc_ is the cathodic peak potential*, E*_pa_ is the anodic peak potential, *E*^θ^ is the standard electrode potential, α is the electron transfer coefficient*,* n is the electron transfer number*, k_s_* is the standard heterogeneous reaction rate constant, *R* is the gas constant*, T* is the thermodynamic temperature*,* and *F* is the faraday constant.

By the slope of the *E*_pa_–ln*υ* curve, α_HQ_ = 0.5826 and α_CC_ = 0.5909 can be obtained.

According to the Randles–Sevcik formula [46] for a quasi-reversible reaction controlled by diffusion:

(6) $$I_{\mathrm{pa}}\;=\;2.69\;\times \;10^{5}\;n^{3/2}\;A\;D^{1/2}\;C_{0}\;\upsilon^{1/2}$$

where *D* is the reactant diffusion coefficient (cm^2^/s)*, A* is the electroactive surface area (cm^2^)*, n* is the electron transfer number, *υ* is the scan rate (V/s), C_0_ is the reactant concentration (mol/cm^3^), and *I*_pa_ is the anodic peak current (A).

With a concentration of 1 mmol/L K_3_Fe(CN)_6_ as the model material (diffusion coefficient *D* = 7.6 × 10^−6^cm^2^/s, n = 1), the relationship between the anodic peak current (*I*_pa_) and scan rates (*v*) is *I*_pa_= 1.09 × 10^−5^ + 0.7959 × 10^−4^ *υ*^1/2^, and the slope is 0.7959 × 10^−4^ = 2.69 × 10^5^ n^3/2^AD^1/2^C_0_. The electroactive surface area of modified electrode can be concluded to be *A* = 0.1073 cm^2^. By the linear relationship of the anodic peak current (*I*_pa_) and scan rates (*v*) on the modified electrode *I*_pa,HQ_ (μA) = 0.4469 + 4.892 *υ*^1/2^ and according to the Randles–Sevcik formula, there is a straight slope 4.892 × 10^−6^ = 2.69 × 10^5^ *n*^3/2^*AD*^1/2^C_0_ (*n* = 2, *A* = 0.1073 cm^2^, C_0_ = 0.1 mmol/L). The diffusivity of HQ in pH = 7 phosphate buffer solution can be obtained as *D* = 3.591 × 10^−7^ cm^2^/s. Similarly, *I*_pa,CC_ (μA) = 0.5529 + 5.646 *υ*^1/2^, and the diffusion coefficient of CC in pH = 7 phosphate buffer solution can be determined as 1.913 × 10^−6^ cm^2^/s.

**Figure 7:**
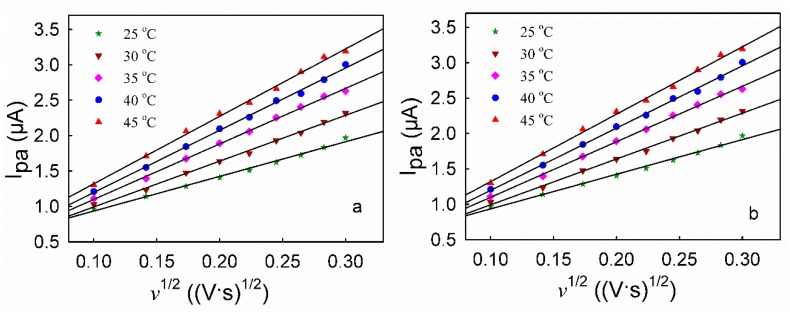
The relationship between *I*_pa_ and *v*^1/2^ of (**a**) HQ and (**b**) CC at different temperatures.

The CV curves of 0.1 mmol/L HQ and 0.05 mmol/L CC on the PGDIL–TiO_2_/Au composite film electrode at different temperatures were tested experimentally, and the relationship between the obtained oxidation peak current (*I*_pa_) and the square root of scan rate (*v*^1/2^) is shown in Figure 7. The values of the diffusion coefficients of HQ and CC (*D*_HQ_ and *D*_CC_, respectively) at different temperatures were calculated according to the diffusion coefficient formula (Table 2). It can be found from the table that both the *D*_HQ_ and *D*_CC_ increased with the increase of temperature, possibly because the increase of temperature led to the increase of kinetic energy of HQ and CC and the decrease of the viscosity of the solution medium, which is beneficial to diffusion.

According to Arrhenius equation [47], the relationship between temperature and diffusion coefficient is in accordance with Equation (7),

(7) $$D=D_{0}e^{-E_{D}/RT}$$

where *D* is the diffusion coefficient when the temperature is *T* (cm^2^/s), and *D*_0_ is the empirical parameter. The formula can be obtained by logarithmic transformation of the formula; i.e., there is a linear relationship between ln*D* and 1/*T*.

(8) $$\ln D=\ln D_{0}-E_{D}/RT$$

**Figure 8:**
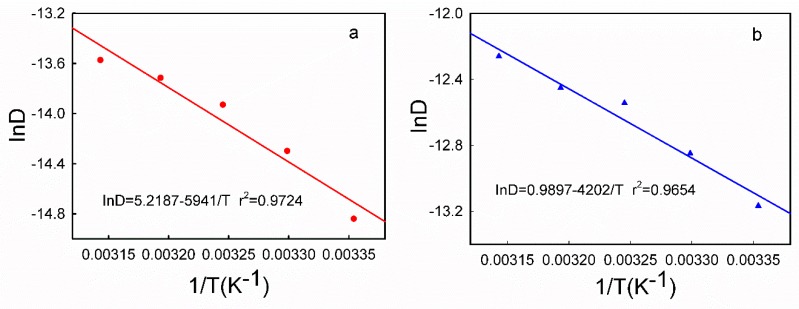
The relationship between ln*D* and (1/T) of HQ (**a**) and CC (**b**).

According to the data of *D*_HQ_ and temperature in Table 2, the fitting equation is ln*D*_HQ_ = 5.2187 − 5941/T (As shown in Figure 8a). As can be seen from Equation (8), the slope of the fitting line is −*E*_D_/R. Therefore, the diffusion activation energy of HQ is *E*_D,HQ_ = 49393 J/mol = 49.39 kJ/mol. Similarly, the diffusion activation energy of CC is *E*_D,CC_ = 34935 J/mol = 34.935 kJ/mol (as shown in Figure 8b).

### 3.5. DPV Analyses of HQ and CC on Different Modified Electrodes

**Figure 9:**
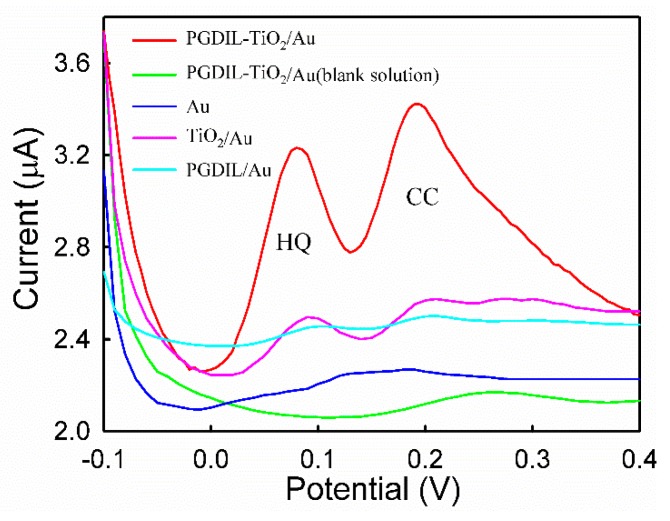
DPV results of 0.1 mmol/L HQ and 0.05 mol/L CC on the PGDIL–TiO_2_/Au, PGDIL/Au, TiO_2_/Au, and Au in pH = 7 PBS at a scan rate of 0.1 V/s.

The electrochemical behaviors of the PGDIL–TiO_2_/Au, PGDIL /Au, TiO_2_/Au, and Au electrodes in the buffer solution containing 0.1 mmol/L HQ and 0.05 mmol/L CC were studied by differential pulse voltammetry (DPV) to investigate the practicability of the PGDIL–TiO_2_/Au electrode. As shown in Figure 9, there was no peak current response in the blank buffer solution in the range of −0.1–0.4 V, which indicates that the buffer solution can be used as the base solution for the electrocatalytic reaction of HQ and CC without impure peak interferences. There was no difference between the anodic peaks of HQ and CC on the bare gold electrode; only a small and wide anodic peak could be observed. The PGDIL/Au and TiO_2_/Au electrodes exhibited respective anodic peaks, but their response sensitivity was low, their current was small, and the two peaks were indistinct, so it was difficult to distinguish them. However, there were two completely separated and responsive anodic peaks of HQ and CC on the PGDIL–TiO_2_/Au electrode. The anodic peak potentials were 0.18 and 0.3 V, which correspond to the oxidation reactions of HQ and CC, respectively. The anodic peak currents of HQ and CC on the PGDIL–TiO_2_/Au modified electrode were 3.22 and 3.42 μA, respectively, and are much higher than those of the PGDIL /Au and TiO_2_/Au electrodes. The results demonstrate that the PGDIL–TiO_2_/Au electrode can effectively improve the electrochemical behaviors of HQ and CC. The anodic peaks of HQ and CC can be completely separated into two sensitive anodic peaks. The peak-to-peak potential difference was 0.12 V, which is slightly higher than the potential reported in the work by [48]. It, therefore, provides a new electrode material for the simultaneous determination of HQ and CC.

**Figure 10:**
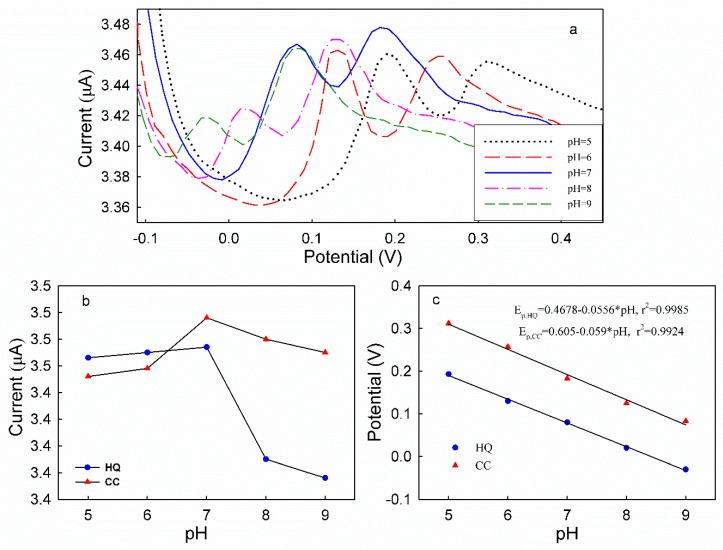
(**a**) DPV results of 0.1 mmol/L HQ and 0.05 mmol/L CC on the PGDIL–TiO_2_/Au electrode under different pHs at a scan rate of 0.1 V/s. (**b**) The relationship between *I*_p_ and pH. (**c**) The relationship between *E*_p_ and pH.

The effects of pH on the electrochemical behaviors of HQ and CC were studied by DPVs. As shown in Figure 10, in the range of pH = 5–9, the anodic peaks of HQ and CC were completely separated. The anodic peak currents increased with the decrease of pH value, and reached the maximum value at pH = 7.0.

The oxidation peak potentials (*E*_p_) of HQ and CC were negatively shifted with the increase of pH values (Figure 10c), and presented good linear relationship with slopes of 0.0556 and 0.059, respectively (*E*_p,HQ_ = 0.4678–0.0556 pH, r^2^ = 0.9985; *E*_p,CC_ = 0.605–0.059 pH, r^2^ = 0.9924). These values are close to the Nernst theoretical value, indicating that H^+^ entered into the electrode reaction, and the number of protons was equal to the number of electrons in the reaction [49]. According to the above conclusion, the number of electron transfers for both HQ and CC during electrochemical oxidation was two, so the redox reactions of HQ and CC were two-electron and two-proton transfer processes, which is consistent with the existing literature [40]. In total, 0.1 mol/L PBS (pH = 7.0) was selected as the support electrolyte for the detection of HQ and CC to obtain high sensitivity and selectivity.

**Figure 11:**
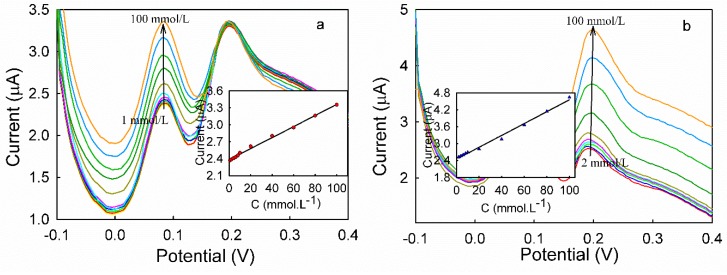
(**a**) DPV results of the PGDIL–TiO_2_/Au in the presence of 0.1 mmol/L HQ with CC at different concentrations (the inset is the calibration curve of the peak current and target concentration). (**b**) DPV results of the PGDIL–TiO_2_/Au in the presence of 0.05 mmol/L CC with HQ at different concentrations (the inset is the calibration curve of the peak current and target concentration). The scan rate is 0.1 V/s.

The selective and simultaneous determination of HQ and CC were performed on the PGDIL–TiO_2_/Au electrode at pH = 7 using DPV. The selective determinations of HQ and CC were carried out by changing the concentration of each isomer separately. Figure 11a,b presents the variations of HQ and CC concentrations in the range of 1–100 μmol/L and 2–100 μmol/L. The results show that the oxidation peak currents are related to the increase of HQ concentration, while the peak currents of CC remain basically unchanged (as shown in Figure 11a). Similarly, the peak currents of CC increased with the increase of the concentration, whereas the peak currents of HQ remained basically unchanged (as shown in Figure 10b). The peak current also had a good linear relationship. The regression equations were *I*_p,HQ_ (μA) = 2.380 + 0.009804 *C*_HQ_ (μmol/L) and *I*_p,CC_ (μA) = 2.380 + 0.009804 *C*_CC_ (μmol/L), respectively. The detection limits (LODs) for HQ and CC were estimated to be 0.23 μmol/L and 0.41 μmol/L (*S*/*N* = 3), respectively.

**Figure 12:**
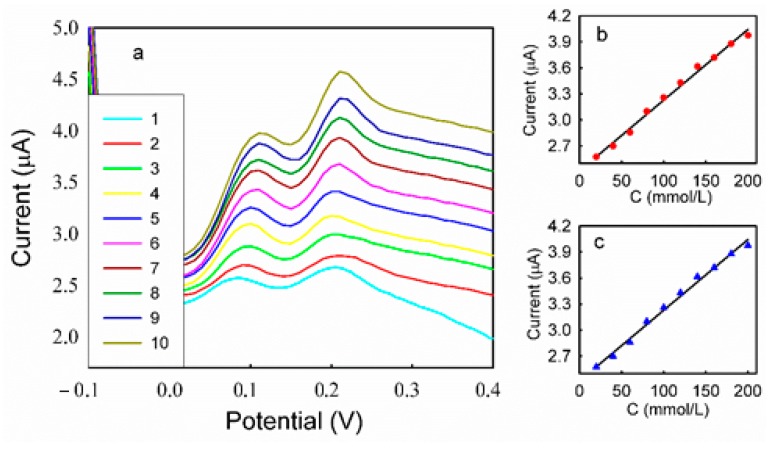
(**a**) DPV results of the binary mixture of CC and HQ at various concentrations on the PGDIL–TiO_2_/Au in pH = 7 PBS. (**b**) The relationship between *I*_pHQ_ and *C*_HQ_. (**c**) The relationship between *I*_pCC_ and *C*_CC_.

Figure 12a displays the DPV results of the binary mixture of CC and HQ at various concentrations. The composition of the binary mixture of CC and HQ is shown in Table 3. The results show two well-defined and separated oxidation peaks for CC and HQ with their respective concentrations as displayed in calibration plots (insets in Figure 12b,c). The regression equations for HQ, and CC are *I*_p, HQ_ (μA) = 3.413 + 0.008152 *C*_HQ_ (μmol/L) (r^2^ = 0.9929), and *I*_p, CC_ (μA) = 2.368 + 0.02179 *C*_CC_ (μmol/L) (r^2^ = 0.9952), respectively. The LODs for HQ and CC were estimated to be 0.17 μmol/L and 0.25 μmol/L, respectively. Therefore, the sensitive and simultaneous determination of HQ and CC are favored without valid interference between them. Thus, the PGDIL–TiO_2_/Au modified electrode is a competitive candidate in simultaneous determination of dihydroxybenzene isomers compared with other modified electrodes [50].

## 4. Conclusions

A novel poly-geminal dicationic ionic liquid-TiO_2_ (PGDIL)-TiO_2_/Au composite electrode was synthesized via the electrochemical polymerization of 1,4-bis(3-(m-aminobenzyl)imidazol-1-yl)butane bis(hexafluorinephosphate) in an electrolyte containing nano-TiO_2_. The SEM results showed that the morphologies of the PGDIL and PGDIL–TiO_2_ powder are similar. Additionally, the PGDIL–TiO_2_ powder has a more uniform and smaller particle size than the PGDIL because the O-vacancies of TiO_2_ can adsorb GDIL molecules to form the core of polymerization, and thus, the polymerization process can be carried out smoothly and slowly. EIS results revealed that the *R*_ct_ value of the PGDIL–TiO_2_ electrode is higher than those of the PGDIL and TiO_2_ electrodes, but its catalytic effect on HQ and CC is the best due to the synergistic effect between TiO_2_ and PGDIL. Furthermore, the DPV method was used to study the simultaneous qualitative and quantitative determination of HQ and CC at the PGDIL–TiO_2_/Au electrode. The results showed that the peak current of each component in the mixture had almost non-interference during electrochemical selectivity determination, and the peak current values were linearly related to their concentrations. The equation for HQ is *I*_p, HQ_ (μA) = 3.413 + 0.008152 *C*_HQ_ (μmol/L), and that for CC is *I*_p, CC_ (μA) = 2.368 + 0.02179 *C*_CC_ (μmol/L). The PGDIL–TiO_2_/Au electrode can be used for simultaneous qualitative and quantitative electrochemical determination of HQ and CC in wastewater by the DPV tests. The PGDIL–TiO_2_/Au electrode is also expected to be used for catalytic oxidation and detection of other organic pollutants containing –OH groups. Further experiments and studies are required to determine whether there are other synergistic effects between TiO_2_ and PGDIL.

## Figures and Tables

**Table 1:** Electrochemical parameters of CC and HQ on different modified electrodes.

Electrode	PGIL-TiO_2_/Au			PGIL/Au			TiO_2_/Au			Au		
*E*/V vs Ag/AgCl	*E*_pa_	*E*_pc_	*∆E*_p_	*E*_pa_	*E*_pc_	*∆E*_p_	*E*_pa_	*E*_pc_	*∆E*_p_	*E*_pa_	*E*_pc_	*∆E*_p_
HQ	0.070	0.030	0.040	0.165	0.065	0.100	>0.225	−0.005	>0.23	>0.175	<−0.05	>0.23
CC	0.235	0.205	0.03	0.249	0.175	0.074	0.234	0.140	0.094	0.244	0.195	0.049

**Table 2:** Diffusion coefficients of HQ and CC at different temperatures.

**HQ**	***T* (°C)**	**25**	**30**	**35**	**40**	**45**
	k × 10^−6^	4.892	6.467	7.840	8.794	9.521
	r	0.9964	0.9982	0.9992	0.9987	0.9984
	D × 10^−7^ (cm^2^/s)	3.591	6.171	8.923	11.05	12.75
**CC**	k × 10^−6^	5.646	6.674	7.245	8.272	9.172
	r	0.9945	0.9964	0.9955	0.9986	0.9990
	D × 10^−6^ (cm^2^/s)	1.913	2.629	3.569	3.910	4.731

**Table 3:** The composition of the binary mixture of CC and HQ.

Binary Mixture	Concentration of HQ (mmol/L)	Concentration of CC (mmol/L)
1	0.02	0.01
2	0.04	0.02
3	0.06	0.03
4	0.08	0.04
5	0.10	0.05
6	0.12	0.06
7	0.14	0.07
8	0.16	0.08
9	0.18	0.09
10	0.20	0.10
